# Measuring Muscle Post-Exercise Oxygen Consumption in Individuals with a Family History of Diabetes

**DOI:** 10.3390/jfmk11010055

**Published:** 2026-01-29

**Authors:** Kevin K. McCully, Olivia Kachappilly, Charlotte Flame, Abheeraj Jain

**Affiliations:** 1 Department of Kinesiology, University of Georgia, Athens, GA 30602, USA; olivia.kachappilly@uga.edu (O.K.); charlotte.flame@uga.edu (C.F.); 2 InfraredRx, Inc., Athens, GA 30602, USA; raj@infraredrx.com

**Keywords:** near-infrared spectroscopy (NIRS), oxidative metabolism, mitochondrial capacity, skeletal muscle, neuromuscular electrical stimulation

## Abstract

**Background:** Post-exercise oxygen consumption (EPOC) contributes to the health benefits of exercise, and changes in EPOC may play a role in the development of diabetes. Near-infrared spectroscopy (NIRS) is a tool used to evaluate muscle metabolism. This study used a novel NIRS-based method of measuring EPOC in the forearm muscles of young adults with and without a family history of diabetes. **Methods:** Fourteen female adults with and without an immediate family history of diabetes were tested. A two-group, one-day design was used with three protocols: ischemic reperfusion, EPOC, and mitochondrial capacity. Muscle oxygen levels were measured with NIRS in the forearm. Blood flow was assessed as the initial reperfusion rate following 5 min of ischemia. EPOC was measured after 60 s of rapid wrist curls with a 2.3 kg weight, followed by measurements every minute for 10 min. Muscle mitochondrial capacity (mVO_2_max) was determined from the recovery rate of muscle metabolism after 30 s of electrical stimulation. EPOC was calculated as the area under the curve of oxygen consumption over 10 min after exercise, subtracting the phosphocreatine contribution calculated from mVO_2_max. Group comparisons were made using *t*-tests with significance at *p* < 0.05. **Results:** mVO_2_max was not different between those with a positive (1.60 ± 0.15 min^−1^) and those with a negative family history (1.45 ± 0.17 min^−1^), *p* = 0.11. Net EPOC was not different between those with a positive (20.0 ± 7.2 O_2_·s) and those with a negative (19.6 ± 11.3 O_2_·s) family history, *p* = 0.94. **Conclusions:** Muscle EPOC minus PCr was calculated after a short, intense bout of exercise. No differences were found in the mitochondrial capacity or EPOC between young healthy individuals with and without a family history of diabetes. This study presents the use of EPOC to evaluate muscle metabolism in populations at risk for diabetes and other related disorders.

## 1. Introduction

Skeletal muscle makes up as much as 40% of the mass of the human body. The energy demands of skeletal muscle at rest, during exercise, and after exercise make important contributions to health. Post-exercise oxygen consumption (EPOC) is a well-studied and important component of the body’s response to exercise [1,2,3,4,5,6]. Typically, EPOC is measured as changes in whole-body oxidative metabolism after physical activity, although recent studies have started to evaluate EPOC in specific muscles (mEPOC) [7,8]. These studies have reported that mEPOC can be as high as fourfold resting metabolism 15 min after exercise has stopped [7,8]. A major metabolic contributor to EPOC is the resynthesis of phosphocreatine (PCr) [2], which is typically completed in 1–3 min [9]. However, other factors such as increased muscle temperature, increased lactate metabolism, return of calcium to the sarcoplasmic reticulum, glycogen resynthesis, and activation of genetic pathways can contribute to the longer-lasting EPOC [2].

Diabetes is a very prevalent chronic medical condition involving impaired glucose metabolism [10,11,12]. Diabetes results in elevated (and reduced) blood sugar levels, which are associated with serious medical complications and increased risk of death. Diabetes can have different causes and is typically classified as Type 1, Type 2, or gestational diabetes. There is some evidence that women are affected by diabetes differently than men, for example, diabetic women have a greater risk of cardiovascular disease than diabetic men [13]. Previous studies have suggested that diabetes (Type 1 and Type 2) can be associated with abnormal or reduced muscle metabolism [2,14,15,16,17,18]. However, diabetes is not always associated with impaired mitochondrial function [19,20]. While muscle mitochondrial capacity is universally reduced in people with motor complete spinal cord injuries [21], the occurrence of diabetes in this population is increased but not uniform [22]. EPOC has been measured in men with metabolic syndrome, although comparisons to men without metabolic syndrome were not made [6]. Several studies have implied that metabolic alterations occur in children whose parents are either Type I or Type 2 diabetics [23,24,25].

The purpose of this exploratory pilot study is to demonstrate a new method of measuring mEPOC, and, specifically, to measure mEPOC separate from the contributions of PCr resynthesis. Near-infrared spectroscopy (NIRS) along with brief periods of ischemia will be used to measure muscle metabolism [26]. Electrical stimulation along with NIRS and brief periods of ischemia will be used to measure muscle mitochondrial capacity (mVO_2_max). This study will present mean values for mEPOC as well as population variance for a relatively homogenous population of young adult females. This study will also test the hypotheses that young adults with an immediate family history of diabetes have different mEPOC and mVO_2_max values compared to young adults without an immediate family history of diabetes.

## 2. Materials and Methods

### 2.1. Participants

Young, healthy females between 19 and 21 years of age were tested. None of the subjects performed regular exercise that used their arm muscles. All subjects performed low-to-moderate levels of leg-based physical activity according to self-report. Participants were divided into two groups based on self-report of the presence or absence of an immediate family history of diabetes. Immediate family history was defined as having at least one biological parent or sibling who is prediabetic or diagnosed with Type 1 or Type 2 diabetes. Family history of gestational diabetes was not included as self-recall by the daughter was not considered reliable. The combination of either Type 1 or Type 2 diabetes was made based on similar symptoms despite different causal mechanisms. Age, height, weight, sex, and adipose tissue thickness (ATT) were recorded. All participants provided written informed consent prior to testing. All procedures were approved by an Institutional Review Board (WCG).

### 2.2. Experimental Design

**Figure 1:**
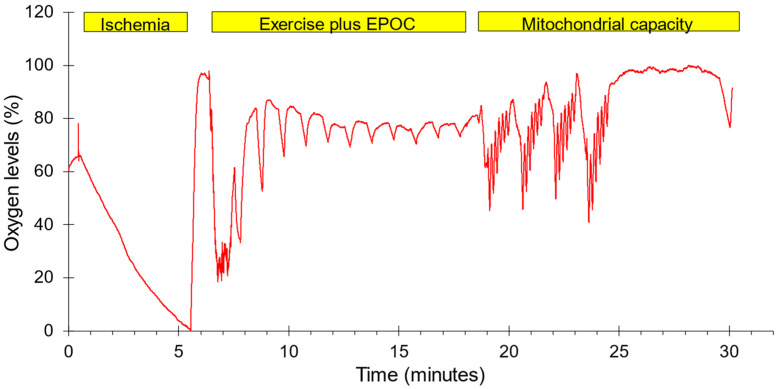
The order of testing and a representative example of oxygen levels during the experimental protocol. Ischemia: five minutes of ischemia followed by reperfusion. Exercise plus EPOC: one minute of strenuous exercise followed by 11 brief ischemic periods to measure the recovery of mVO_2_. Mitochondrial capacity: four bouts of 30 s of NMES followed by six brief ischemic periods along with a final ischemic period to measure the rate of recovery of mVO_2_. Oxygen levels were normalized to the lowest and highest values during the entire protocol.

A two-group, one-day experimental design was conducted. Each subject completed three test protocols in one test session on either the dominant (N = 7) or non-dominant forearm (N = 7). The order in which the tests were performed along with representative oxygen levels are shown in Figure 1. The forearm muscles were chosen to represent muscles that are not typically used during recreational physical activity. Previous studies have shown that mitochondrial capacity is lower in arm than leg muscles, and that leg-based physical activity increases leg and not arm muscle mitochondria [27,28]. This was felt to reduce the need to account for potential differences in physical activity level between subjects. The protocols included 5 min of ischemia followed by reperfusion to provide a calibration scale for the NIRS signals, a post-exercise oxygen consumption protocol lasting 10 min, and a standard mitochondrial capacity protocol. Muscle oxygenation was measured using a Train.Red Plus NIRS device (TrainRed Plus, TrainRed, Elst, The Netherlands), with a source detector separation distance of up to 4 cm and a sampling rate of 10 Hz. The NIRS probe was placed on the belly of forearm flexor muscles, approximately 25% of the distance from the elbow to the wrist and 45 degrees laterally from the ventral side of the forearm. The probe was lightly secured to the arm with pre-wrap tape. A blood pressure cuff (Hokanson 10 cm) was placed on the upper arm of the tested limb. The cuff was connected to a Rapid Cuff Inflation system (E20, Hokanson, Milwaukee, WI, USA, and a California Air Tools 15-gallon air compressor/tank). Participants were seated with their arms supported at heart level. All subjects were told to avoid caffeine, alcohol, and strenuous activity for 24 h prior to testing. Each subject was placed on a padded table, supine, with both legs positioned straight. The left foot was secured in a stabilization holder on the padded table to limit motion artifacts in the NIRS signal. The knee was supported by a cushion. Measurements to find muscle oxygen levels were taken prior to and immediately following the electrical stimulation protocol. The experimental set up for the NIRS measurements used a continuous-wave NIRS device (TrainRed Plus). The NIRS data were collected at 10 Hz.

### 2.3. Ischemic Reperfusion Test Procedure

To measure a calibration range for the NIRS signals and to measure blood flow as the initial oxygen recovery during reperfusion, the cuff was rapidly inflated to super systolic pressures (>220 mmHg) for 5 min, followed by rapid deflation of the cuff and 60–90 s of recovery time [29]. The minimum oxygen level during ischemia was assumed to be zero % oxygen, and the highest value during reperfusion was assumed to be 100% oxygenated. All NIRS values are presented using this scale. Upon cuff release, the initial reperfusion rate was measured as the fastest slope during the first 20% of the recovery of oxygen levels. A time to half recovery (T_1/2_) was also measured.

### 2.4. Post-Exercise Oxygen Consumption (mEPOC) Test Procedure

Subjects performed 60 s of rapid wrist curls (~1 per second) using a 2.3 kg dumbbell. The subjects were verbally encouraged to give maximal effort and to continue to perform rapid, full-range-of-motion wrist curls during the entire 60 s. Subjects were given verbal instructions on how to perform the exercise, but had no prior experience performing the exercise. Immediately following the cessation of exercise, a series of cuff occlusions was conducted to measure oxygen recovery. The cuff was inflated for 15 s, followed by 45 s of rest, and this cycle was repeated 11 times. The slope of the decline in muscle oxygen levels was used as a relative measure of muscle metabolism (mVO_2_). Total mEPOC was calculated by adding the individual mVO_2_ values over the ten minutes post-exercise. PCr mEPOC was calculated as the predicted muscle metabolism due to PCr resynthesis using the rate constant from the mitochondrial capacity tests. Prior studies have shown excellent agreement between the recovery rate of PCr from ^31^P MRS and the recovery of mVO_2_ using NIRS [30]. The net mEPOC was then calculated as the difference between the total mEPOC and the PCr mEPOC. mEPOC values were also calculated as metabolic equivalents (METs) by dividing the mVO_2_ values by resting mVO_2_.

### 2.5. Mitochondrial Capacity Test Procedure

The rate of recovery of muscle metabolism after exercise was used as an index of muscle oxidative capacity [31]. mVO_2_max was assessed using 30 s of NMES followed by a series of brief cuff occlusions which consisted of six cycles of cuff inflation and release (5 s on, 5 s off) to measure oxygen recovery kinetics [32]. This procedure was repeated four times. NMES consisted of biphasic 250 μs twitch contractions at 4 Hz. Current intensity was adjusted to produce vigorous visible muscle contractions. Five minutes after the last cycle of cuff inflations, a final 30 s cuff occlusion was taken to obtain a final mVO_2_ value. mVO_2_max was calculated as the rate constant of exponential recovery of the mVO_2_ values using the equation listed below:

mVO_2_ = End + Delta * e^(-T*Rc)^

where mVO_2_ is the slope of the oxygen signal during cuff ischemia, End is the final mVO_2_ slope five minutes after the last recovery slope, Delta is the difference in mVO_2_ values from the first slope and the last slope, T is time since the start of recovery at NMES, Rc is the rate constant. Each of the four recovery curves were analyzed separately. The mVO_2_ values for the four curves were also combined and fit to the recovery equation to produce average values. These average values were used for statistical comparisons.

### 2.6. Biometric Data

Age, skin color, hair color, hair thickness, height, weight, and body mass index (BMI) were collected. Height and weight were used to calculate BMI. ATT was measured at the end of the testing session at the site of the NIRS optode with ultrasound (Butterfly IQ+, Butterfly Network Inc., Burlington, MA, USA).

### 2.7. Analysis

Values are presented as means with standard deviations. Unpaired *t*-tests were performed for comparisons. Data were evaluated for normal distributions. Significance levels (*p* values) were set as 0.05 for all comparisons. Sample size calculations were based on prior mVO_2_max measurements [33]. Using an unpaired *T*-test with a mean value of 1.6 min^−1^ and a standard deviation of 0.25 min^−1^, we would be able to detect a 25% difference in mVO_2_max with a power of 0.80 with a sample size of 7 per group.

## 3. Results

### 3.1. General Results

Participant characteristics are shown in Table 1. Data from all 14 subjects were used for analysis. There were no differences between groups except for in height, where the Negative group was 6 mm taller on average. In six subjects, the total EPOC values were not extended to 10 min (durations were 7, 7, 8, 9, 9, and 9 min). For measurement of EPOC over 10 min, the total EPOC values were extended using linear extrapolation. Linear extrapolation was considered appropriate as the later parts of the change in mVO_2_ values approximate a linear curve. No changes in significance were seen between comparisons using the 10 min extrapolated values and when using 7 min values (where a complete data set was available). Using normalized NIRS values, reactive hyperemia T_1/2_ values, mVO_2_max, and net mEPOC values did not correlate with either skin color or ATT levels (all *p* > 0.05). Reactive hyperemia initial slope values did correlate with ATT values (*p* = 0.02), although not with skin color. Interestingly, greater ATT levels were associated with higher RH values.

### 3.2. Ischemic Reperfusion Test

The absolute SmO_2_ magnitude (max. minus min. signal) was not different between groups: 34.8 ± 5.5%O_2_ and 36.6 ± 13.2%O_2_ for the Positive and Negative groups, respectively, *p* = 0.75. Normalized resting mVO_2_ values were not different between groups: 0.35 ± 0.08%O_2_/s and 0.36 ± 0.07%O_2_/s for the Positive and Negative groups, respectively, *p* = 0.91. Normalized reactive hyperemia blood flow after 5 min of ischemia was not different between groups: 6.46 ± 1.79%O_2_/s and 6.26 ± 2.42%O_2_/s for the Positive and Negative groups, respectively, *p* = 0.88.

### 3.3. Mitochondrial Capacity Test

**Figure 2:**
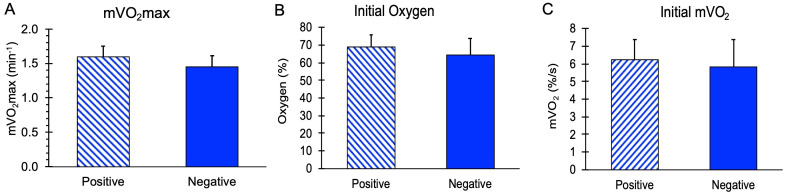
Mitochondrial capacity variables for people positive or negative for a family history of diabetes. (**A**) Muscle mitochondrial capacity (mVO_2_max). (**B**) Initial post-NMES mVO_2_ values. (**C**) Initial oxygen levels during the mitochondrial capacity test. Values are means with SD. *p* > 0.05 for all comparisons between groups.

mVO_2_max values, along with initial mVO_2_ and initial Oxygen levels during the recovery measurements, are shown in Figure 2. There were no significant differences in these values between the subjects with and without a family history of diabetes (*p* = 0.11, 0.59, and 0.36 for mVO_2_max, initial mVO_2_, and initial oxygen, respectively). The stimulation ratio (NMES/resting mVO_2_) was not different between the Positive (10.2 ± 1.8) and Negative (9.3 ± 2.1) groups, *p* = 0.37. Using a group mean for mVO_2_max of 1.52 min^−1^ and a group standard deviation of 0.17 min^−1^, a difference of 16% could have been detected in this study.

### 3.4. Post-Exercise Oxygen Consumption (mEPOC) Test

**Figure 3:**
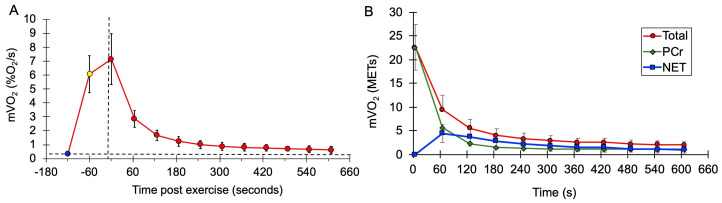
(**A**) Average mVO_2_ rate values at rest (blue circle), at the end of the NMES from the mitochondrial capacity test (yellow circle), and after voluntary exercise (red circles). Vertical dashed line is the start of recovery. The horizontal dashed line represents the initial resting metabolism. (**B**) Average values during EPOC for total metabolism (red circles and line), PCr recovery (green triangles and line), and net EPOC (blue squares and line). Error bases are SD.

mVO_2_ values after exercise during the mEPOC protocol for all subjects combined are shown in Figure 3. Values are presented as normalized mVO_2_ values (Figure 3A), as well as calculated METs values (Figure 3B).

**Figure 4:**
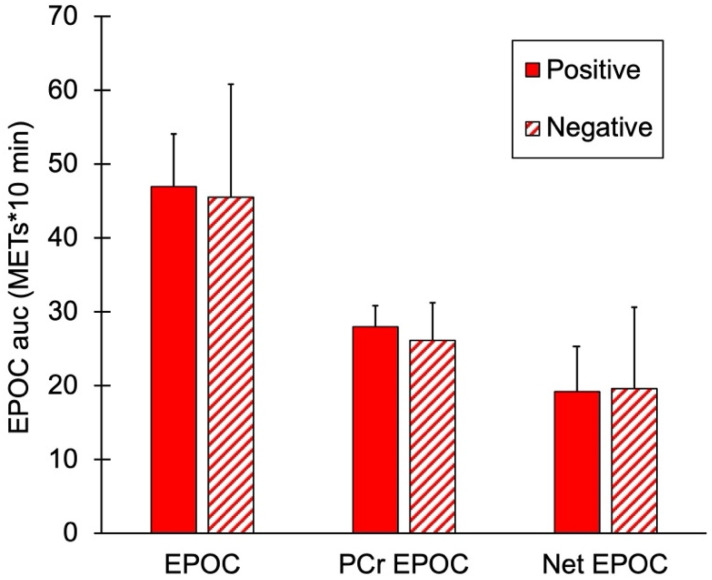
Muscle post-exercise oxygen consumption (mEPOC) variables for people positive or negative for a family history of diabetes. Total mEPOC values, mEPOC calculated from PCr recovery, and Net mEPOC as total minus PCr values. Values are means with SD. *p* > 0.05 for all comparisons between groups.

mEPOC AUC values for the two groups are shown in Figure 4. There were no significant differences in total EPOC, PCr mEPOC, or net mEPOC values between the subjects with and without a family history of diabetes (*p* = 0.74, 0.41, and 0.93, respectively).

## 4. Discussion

This study presents a protocol for determining post-exercise oxygen consumption (EPOC) in specific muscles rather than the whole body. Following a minute of voluntary exercise, muscle metabolism was on average 4.5 times higher than resting, although most of this increase was during the first few minutes. The value of 1.4 times resting muscle metabolism after 10 min was consistent with prior studies of muscle metabolism after 30 s of electrical stimulation but less than that reported after performing a maximal cycling exercise [7,8]. The previous studies reported post-exercise metabolism at one time point (either 5 or 15 min) [7,8]. This study presented a time course of recovery of muscle metabolism over 10 min. It was clear that, after 7 min, muscle metabolism was declining to resting levels in a slow and linear fashion. This suggests that using the 10 min time point was adequate to characterize the metabolic response to exercise.

A key aspect of this study was the determination of post-exercise oxygen consumption due to phosphocreatine resynthesis and the subtraction of this contribution to obtain EPOC due to factors other than PCr resynthesis [2]. The contribution of PCr resynthesis was determined from the calculation of the recovery rate from the mitochondrial capacity test. This was not expected to be the same as it would be if it was determined from actual PCr concentrations measured with phosphorous magnetic resonance spectroscopy (^31^P MRS). That is because strenuous exercise can lower muscle pH [35]. The increase in hydrogen ions offsets the creatine kinase equilibrium such that measured PCr concentrations will not reflect changes in muscle metabolism. The oxygen consumption that is independent of PCr resynthesis may be related to the health benefits of EPOC. Using this approach, approximately 60% of the total EPOC over 10 min was due to PCr resynthesis. What would happen with other types, intensities, or durations of exercise remains to be determined. Increased intensity and duration of exercise has been shown to increase EPOC [6]. Initial studies have suggested that increased muscle temperature and lactate levels are a major contributor to EPOC [3]. It is not clear if the duration of exercise used in this study as well as the small size of the muscles exercised were enough to raise muscle temperature enough to produce significant amounts of EPOC. And it is not clear if the temperature response is related to muscle temperature or the whole-body response to increased temperature. Lactate levels may well have risen with the exercise used in this study, although lactate levels were not measured.

The initial comparison performed in this study was with young adults with an immediate family history of diabetes compared to young adults without an immediate family history. We did not find any evidence of metabolic differences between these two groups. This is in contrast to previous studies that have suggested metabolic alterations in children whose parents are diabetic [23,24,25]. One reason for the difference between this study and the previous studies is that a family history of diabetes is often associated with childhood obesity [23]. The subjects in this study were normal weight and there was no difference in weight between the subjects with and without a family history of diabetes. A limitation to our study was the use of self-report of immediate family history of diabetes. This may not be precise enough to identify genetic precursors of diabetes.

Several novel aspects of this study include the use of all women subjects and the reporting of skin color. Skin color is rarely reported in studies that evaluate skeletal muscle oxygen levels using NIRS [36]. This is despite the clear impact of skin color on NIRS signal intensity [37]. The biggest influence on NIRS signals has been reported to be ATT values [38]. This study evaluated the forearm muscles that had relatively low ATT levels (~5 mm). Thus, high-quality signals were obtained in this study. The evaluation of only female subjects was also relatively novel. Potential differences in EPOC have been reported between men and women [2].

This study used normalized values of SmO_2_ to make its comparisons. Five minutes of ischemia has been shown to produce near-zero levels of oxygen concentrations, providing a solid minimum value for oxygen levels. During reactive hyperemia, there is a greater increase in blood flow than muscle metabolism, resulting in an overshoot of oxygen levels. While this is perhaps not a 100% oxygen saturation condition, it does provide a consistent point for calibration purposes. Prior studies have suggested that deoxygenated signal might be a better point to use to determine oxygen levels [39]. However, calibration, as shown in this study, has been reported to reduce differences in NIRS slope measurements in populations that differ in ATT and other conditions [40]. In this study, normalizing the NIRS signals using the physiological calibration resulted in no effect of skin color and ATT on the outcome variables, except for in the case of RH slope vs. ATT.

This study has several limitations. These include the use of left and right arms in both groups. While each group had equal numbers of dominant and non-dominant arms, this could have introduced some added variance to the study. Analysis of dominant and nondominant arms across both groups revealed no differences in any of the variables. There was a small absolute difference in mVO_2_max between the dominant and non-dominant arms (~6%), but this was not statistically significant. A prior study found about a 10% difference between arms with a sample size of 17 per group [33]. Thus, the use of different arms was unlikely to influence the main outcomes of this study. Based on the sample variability in this study, future studies will need to use much larger samples sizes for measurements of reactive hyperemia and mEPOC. Another potential limitation to the study was the use of untrained subjects who were unfamiliar with the wrist curl exercise performed in this study. We intentionally chose to use naive subjects in this way; however, subjects with experience in the protocol may have exercised more vigorously and may have had greater mEPOC and net mEPOC values. Future studies are needed to evaluate this hypothesis. This study also demonstrated the method of evaluating mEPOC, PCr mEPOC, and net mEPOC. Future studies will be needed to evaluate potential contributors to mEPOC, including documenting changes in muscle pH, evaluating the contribution of exercise-induced muscle injury, sustained hypoxia, and glycogen depletion. This study also used self-report of a family history of Type 1 or Type 2 diabetes. Evaluation of potential differences associated with family history is of interest, particularly if it helps predict future diabetes. However, a stronger comparison of the influence of diabetes on muscle function could have used actual diabetic subjects and recorded differences in plasma glucose and insulin levels.

## 5. Conclusions

In conclusion, this study reports a novel method of evaluating local muscle EPOC during the 10 min following exercise. Muscle EPOC was calculated as the total mEPOC and the mEPOC not related to PCr resynthesis. The method was demonstrated in two groups, young female adults with and without a self-reported family history of diabetes. While no differences were found in this pilot study with respect to family history, future studies can further test this hypothesis. The measurement of net mEPOC has the potential to increase our understanding of the contributions of mEPOC to muscle and whole-body health.

## Figures and Tables

**Table 1:** Physical characteristics of participants.

Group	Age, Years	Height, Meters	Weight, Kilograms	BMI kg/m^2^	ATT mm	Skin Color, 1 to 5
Positive	20.3	1.59	52.7	20.9	5.2	2.4
	(0.5)	(0.04)	(3.7)	(1.2)	(0.9)	(0.5)
Negative	20.0	1.65	55.5	20.4	5.1	2.9
	(0.6)	(0.05)	(6.9)	(1.7)	(1.1)	(1.1)
*p* value	0.34	0.04	0.36	0.55	0.73	0.36

Values are expressed as means (SD). ATT, adipose tissue thickness. Positive indicates an immediate family history of diabetes. Negative indicates no immediate family history of diabetes. ATT is adipose tissue thickness over the tested muscle measured using ultrasound. Skin color is estimated based on the Fitzpatrick scale [34].

## Data Availability

The raw data supporting the conclusions of this article will be made available by the authors on request.
